# Toner’s Dictionary of Elevations

**Published:** 1874-05

**Authors:** 


Dictionary of Elevations and Climatic Register of the United States; Containing, in addition to Elevations, the latitude, mean annual temperature, and the total annual rain-fall of many localities; with a brief introduction on the orographic and other physical peculiarities of North America. By J. M. Toner, M.D., New York: D. Van Nostrand, publisher, 23 Murray and 27 Warren streets, 1874. Octavo; Paper, pp. 124. Price, $3.00.

In our November number, we called attention to this promised work of Dr. Toner, and extracted from the advance sheet sent us, a few statistical items which interested us at the time. The completed work before us fulfills the promise then given of a rare and valuable collection of statistical figures, nowhere else obtainable. We have at our command no means of testing, satisfactorily, the accuracy of Dr. Toner's figures or the comparative freedom from typographical errors, but we feel that the patient care and assiduity which are so characteristic of the author, are ample guarantee upon these points. In the mass of significant characters before us lies ample texts for a thousand medical discourses which might be elaborated with interest and profit. We trust there will be found many to engage in this work of elaboration.

